# Establishment of a value assessment framework for orphan medicinal products in China

**DOI:** 10.1186/s13023-024-03393-6

**Published:** 2024-10-20

**Authors:** Handong Chen, Yuliang Xiang, Xian Tang, Ming Hu

**Affiliations:** 1 West China School of Pharmacy, Sichuan University, 17 Renmin South Road (3rd Section), Chengdu, China; 2 School of Public Health, Fudan University, Dong’an Road, Shanghai, China

**Keywords:** MCDA, Value assessment framework, Criteria, Orphan medicinal products, EVIDEM

## Abstract

**Background:**

This study aimed to establish a suitable value assessment framework for orphan medicinal products in China based on the multi-criteria decision analysis (MCDA) method.

**Methods:**

First, a draft framework of the MCDA criteria was built based on a systematic literature evaluation and the EVIDEM framework tools. Second, stakeholder groups were formed and expert opinions were collected through the brainstorming and expert consultation methods. Third, from the perspective of stakeholders, the five-point weighting method and a two-step percentile distribution method were employed to weigh the quantitative criteria in the framework for orphan drug value evaluation. Meanwhile, from the public perspective, a survey was conducted on a sample of 71 people to obtain the scoring scale of the framework for orphan drugs through a two-step percentile distribution method. Finally, based on the synthetization and comparison of all evidence and methods, we developed the framework criteria and scoring scale for the orphan medicinal products.

**Results:**

Combined with the stakeholder selection and suggestions in the stakeholder workshop, the framework criteria for the evaluation were constructed based on China’s national conditions, which included 11 quantitative and 8 qualitative criteria. The two-step percentile distribution method was selected as the weighting method.

**Conclusions:**

MCDA is feasible for the value assessment of orphan drugs in China and can be used as a supplementary tool for drug access decisions in medical insurance. It is suggested to further improve the value assessment framework of orphan medicinal products, scientifically evaluate the MCDA framework weighting method, explore a framework system suitable for China’s national conditions.

**Supplementary Information:**

The online version contains supplementary material available at 10.1186/s13023-024-03393-6.

## Background

Rare diseases, also known as “orphan diseases”, are characterized by low incidence rates, but they also have high mortality and disability rates that seriously negatively impact the quality of life of patients and lead to a heavy societal and economic burden [1]. Although the prevalence of rare diseases is low, the number of patients with such diseases is high. It is estimated that the global number of patients with rare diseases is approximately 400 million [2], with approximately 27–36 million in the EU [3], 2,500–3,000 in the United States [4], and 49–82 million in China, and these values increase by more than 200,000 patients every year [5].

Orphan medicinal products (OMP) are used to prevent, diagnose, and treat rare diseases [6]. Due to the large heterogeneity of rare diseases and their ambiguous pathogeneses as well as small populations, it is difficult to develop orphan drugs with small market capacities. Therefore, the high research and production costs of orphan drugs lead to high market pricing, making it difficult to obtain cost-effective results that can be accepted by medical insurance decision departments using conventional economic evaluation methods [7, 8]. Currently, the main problems faced by orphan medicinal products are low market accessibility, poor access to medical insurance, and poor affordability in the world [9, 10]. Therefore, a comprehensive evaluation of the value of orphan drugs and the provision of a basis for decision-making departments, such as medical insurance, is of concern in both international and domestic fields.

Health technology assessments, cost-effectiveness analyses, and budget impact analyses are currently the main methods of evidence required for healthcare and health decision-making internationally. However, there are many difficulties in the health technology assessment of orphan drugs, such as the drugs’ extremely low prevalence, lack of patients, complex pathogenesis, and efficacy judgement methods, high disease heterogeneity, long courses, and difficulties in adopting a strict randomized controlled study design; orphan drugs often have little clinical evidence of low quality. Therefore, many countries have relaxed their data requirements and have begun considering societal value criteria for orphan drugs when using health technology evaluation methods to assess the value of orphan drugs [11].Currently, the construction of a reasonable drug value assessment framework for rare diseases and medical insurance access evaluation standards is the focus of decision-making departments and researchers in various countries [12, 13].

Multi-criteria decision analysis (MCDA) is increasingly used as an applicable method to assess the value of orphan drugs [14–16] and employed to support decision making on multiple and conflicting criteria [17]. It can decompose a decision into many criteria, rank the individual criteria by importance, and define the influence of each criterion on the decision and its relative importance [18]. Finally, the existing information is used to evaluate the decision-making plan and assist in improving the consistency, transparency, and rationality of decision making [19].

Based on the MCDA method, this study aimed to establish a value assessment framework suitable for orphan drugs in China and explored the ideas and feasibility of comprehensively to assess the value of orphan drugs and medical insurance access decisions across multiple dimensions.

## Methods

### Building the initial framework of the criteria

Prior to commencing this study, a qualitative systematic evaluation was conducted to collect the value assessment criteria of orphan drugs [20]. Three domains and fourteen criteria were extracted using a SPIDER model, and then further literature review showed that the EVIDEM framework was the most frequently used tool in the MCDA studies of orphan drugs, and the criteria we developed had a high overlap with the EVIDEM framework. Therefore, after that we chose the EVIDEM framework as the base to establish an orphan drug value assessment framework in China through survey and justification in this study.

The EVIDEM framework is an open-source, collaborative-development MCDA framework designed to assess the overall value of medical interventions. It can be used to compare various interventions across disease areas to prioritise interventions. The framework was tested and implemented in various real-world decision-making settings [21, 22]. The orphan drug value assessment framework was first proposed by Goetghebeur et al., [23] and includes thirteen quantitative criteria and seven qualitative criteria according to the MCDA methodology principle of non-redundancy, independence, manoeuvrability, and completeness. In Catalonia, Giabert [24] conducted a validation of the orphan drug assessment using the EVIDEM framework and found that the EVIDEM-MCDA framework is useful and feasible for the evaluation and decision-making on orphan drugs. Therefore, our study utilized the EVIDEM (V.10) framework criteria, which were translated into local language and adapted to the medical insurance policy in China and the unique characteristics of orphan drugs. The inclusion of each criterion was comprehensively evaluated from an ethical perspective, resulting in the initial version of the assessment tool (see Appendix 1).

### Forming of the criteria framework

In this study, a total of 15 stakeholder experts were invited as members of the stakeholder expert group (see Appendix 2) and the inclusion criteria are detailed (see Appendix 3). Members of the expert group were well-known researchers or practitioners in the fields of medical insurance, medical care, health economics, and rare diseases in China. Due to the coronavirus diseases (COVID-19) pandemic, we conducted an online workshop in December 2021 through Tencent Conference^®^. The workshop process was as follows: (1) The research team discussed with relevant experts the current status of MCDA and value assessment of orphan drugs in China and internationally; (2) the research team introduced the background, methods, and implementation process of the project to the relevant experts; (3) the stakeholder experts were invited to discuss the selection of criteria in the initially proposed value framework of orphan medicinal products; (4) the stakeholder experts were required to complete an online survey questionnaire immediately, which involved selecting and supplementing the preliminary framework criteria for this study. The research team identified the criteria that needed to be retained within the framework and the new criteria that needed to be added to the framework. (5) Finally, the research team summarised and organized the discussion opinions of the stakeholder experts, modified the research framework by referring to the opinions and the selection results, and created a scoring scale that incorporated the stakeholders’ expert opinions and relevant operational conditions. An orphan drug value criteria framework was developed.

### Weighting for orphan medicinal products based on MCDA

To compare the weight differences of the criteria from different perspectives, we conducted the weighting from the perspective of stakeholders and the public, respectively. A second stakeholder expert workshop was held in April 2022, where the research team invited the stakeholder experts to weight the criteria in the value assessment framework of orphan drugs, based on their perception of the level of importance of each criterion. In order to select the most appropriate method of weighting, we used the five-point weighting and two-step percentile distribution method seperately. For five-point weighting method, each stakeholder expert assigned a relative weight to each criterion using a simple 5-point scale (1 = lowest relative importance; 5 = highest relative importance). The questionnaire is presented in Appendix 4.1. For the two-step percentile distribution method, the stakeholder experts first assigned 100 points to the five first-level domains of the quantitative framework, followed by 100 points between the second-level criteria under each domain, thus obtaining the relative weight of each criterion. The questionnaire is presented in Appendix 4.2.

Three respondents of the public were randomly selected for the pilot study in May 2022, at Sichuan Province, and they were interviewed about their perception after completing the survey. During the interview, respondents were asked to explain their understanding of the criteria and the reasons for their importance scores. After the pilot study, some minor changes were made to the format and wording of the questionnaire. Finally, four trained investigators were divided into two groups, with two investigators assigned to conduct face-to-face interviews for one respondent each time, and respondents were selected by random non-probability sampling. Through the questionnaire, we aimed to know the preferences of the value framework for orphan medicinal products from the societal perspective.

From the perspective of the public, according to the revised quantitative part of the value assessment framework, a questionnaire on the preference of criteria of the value assessment framework for orphan medicinal products was conducted. Considering the principle that the sample size is 5–10 times of the scale items [25], 50 samples were expected to be included and in order to ensure the effective number of samples, 60 samples were planned to be collected. To make the survey data representative, quota standards were set according to the distribution of gender, age and education level in China Statistical Yearbook (2021), see Appendix 5.

By using the two-step percentile distribution method, the respondents first assigned 100 points to first-level domains of the modified EVIDEM framework, followed by 100 points assigned among the second-level criteria under each domain, thus obtaining the relative weight of each criterion. The criterion weight = (domain score mean / 100) * (criterion score mean / 100) * 100%. The weights, standard deviation, and coefficient of variation were calculated by SPSS 17.0^®^.

### Data analysis

All data analyses were performed using Microsoft Excel 2019^®^ and SPSS17.0^®^. The sum of the criteria weights was normalised to 1. For the five-point weighting method, the relative weight of each criterion was divided by the sum of all the criteria. For the two-step percentile distribution method, the relative weight of each criterion was its domain score multiplied by the score of its local criterion and normalised to 1.

Reliability evaluation: The degree of coordination of the expert opinions was expressed by the CV and W. The following formula was used: CV = standard deviation (S)/mean value [26] (M). The Kendall W coefficient (W) [27] indicates the extent of agreement among raters in the ranking of items. Its value ranges between 0 and 1; the larger the value of W, the higher the degree of agreement or consensus among different opinions [28]. The calculation formula is as follows:

$$\:\text{W}=\frac{12}{{\text{m}}^{2}\left({\text{n}}^{3}-\text{n}\right)-\text{m}\sum\:_{\text{i}=1}^{\text{m}}{\text{T}}_{\text{i}}}\sum\:_{\text{i}=1}^{\text{n}}{{\text{d}}_{\text{j}}}^{2}$$

Notes: where, “m” is the total number of participants, “n” is the number of lists, “T_i_” is the correction coefficient, and “d_j_” is the value of the evaluation levels of “m” participants for the “j” lists minus the arithmetic average of the evaluation levels for all criteria. It is generally believed that the coordination coefficient (W) < 0.2 indicates a poor degree of consistency, whereas a degree of consistency between 0.2 and 0.6 is considered moderate [29–31], between 0.6 and 0.8 is considered strong, and between 0.8 and 1.0 is considered very strong.

For questionnaires collected from the public, Excel 2019^®^ was employed to input data, and SPSS 17.0^®^ was used to conduct data processing and descriptive analysis. The coefficient of variation (CV) of the weight was calculated to indicate the coordination degree of the public preference for the importance of the criteria.

## Results

### Building results of the value assessment framework for orphan medicinal products based on MCDA

In the first workshop, 10 representatives out of the 15 experts attended and selected the criteria in the value assessment framework for orphan drugs to be constructed in this study. The quantitative criteria selection vote results for the core model of the value assessment framework are presented in Appendix 6, and the qualitative criteria selection vote results for the contextual tools of the value assessment framework are presented in Appendix 7. During the online workshop, the experts proposed 9 opinions and suggestions for this framework, as shown in Appendix 8.

After the online workshop, the experts were asked to select the criteria and determine the criteria scoring scale based on a summary of the workshop through an online questionnaire. The results of the online questionnaire by the stakeholders are shown in Appendix 9. Combined with the stakeholder experts’ opinions in the first workshop and the online questionnaire, a revised orphan drug value assessment criteria framework was formed. It consists of 11 quantitative and 8 qualitative criteria, as detailed in Table 1.

**Table 1:** Criteria framework for value assessment of orphan medicinal products

Domains	Criteria
**Quantitative criteria of the value assessment framework (core model)**	
Need for drugs	Disease severity
	Unmet needs
Comparative outcomes of drugs	Comparative effectiveness
	Comparative safety / tolerability
	Comparative patient-perceived health / patient-reported outcomes
Type of benefit of drugs	Type of therapeutic benefit
Economic consequences of drugs	Comparative cost consequences–cost of drugs
	Comparative cost consequences–other medical costs
	Comparative cost consequences–non-medical costs
Evidence about drugs	Quality of evidence
	Expert consensus / clinical practice guidelines
**Qualitative criteria of the value assessment framework (contextual tool)**	
Normative contextual criteria	Mandate and scope of the healthcare system
	Population priorities and access
	Common goal and specific interests
Feasibility contextual criteria	System capacity and appropriate use of the intervention
	Government objectives and policy priorities^*^
	Aid program sustainability^*^
	Technological innovation^*^
	Affordability of medical insurance funds

Note: *: source of this criteria from the Stakeholders’ opinion, otherwise from the EVIDEM framework

### Weighting results of the value assessment framework for orphan medicinal products based on MCDA

Thirteen representatives out of 15 stakeholder experts attended and assigned weights to the criteria in the second workshop. Seventy six individuals engaged in the study and assigned weights to the criteria.

**Fig. 1:**
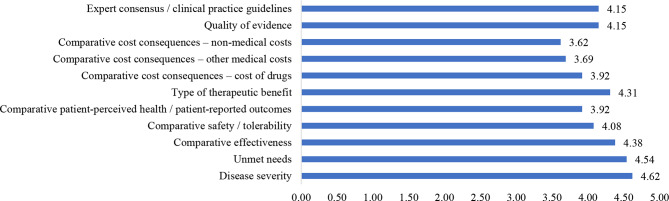
Results of the weighting based on the five-point weighting method

From the perspective of stakeholders, the weighting results measured using the five-point weighting for each criterion in the framework are shown in Fig. 1.

**Fig. 2:**
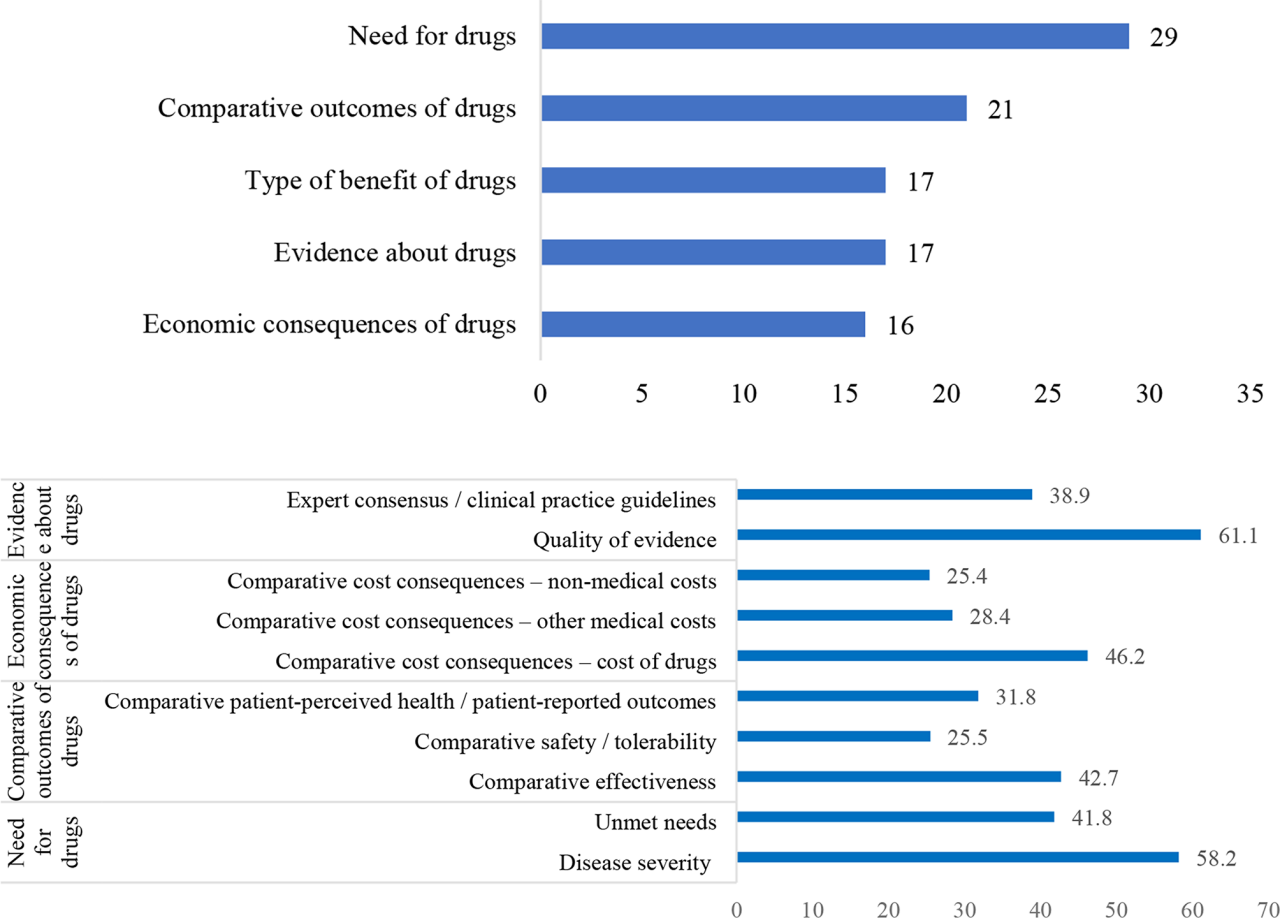
Weighting results based on the two-step percentile distribution method. Note: top: weight allocation results for the first-level domains; bottom: weight allocation results for each criterion

From the perspective of stakeholders, the weighting results measured using the two step percentile distribution methods for each criterion in the framework are shown in Fig. 2.

**Fig. 3:**
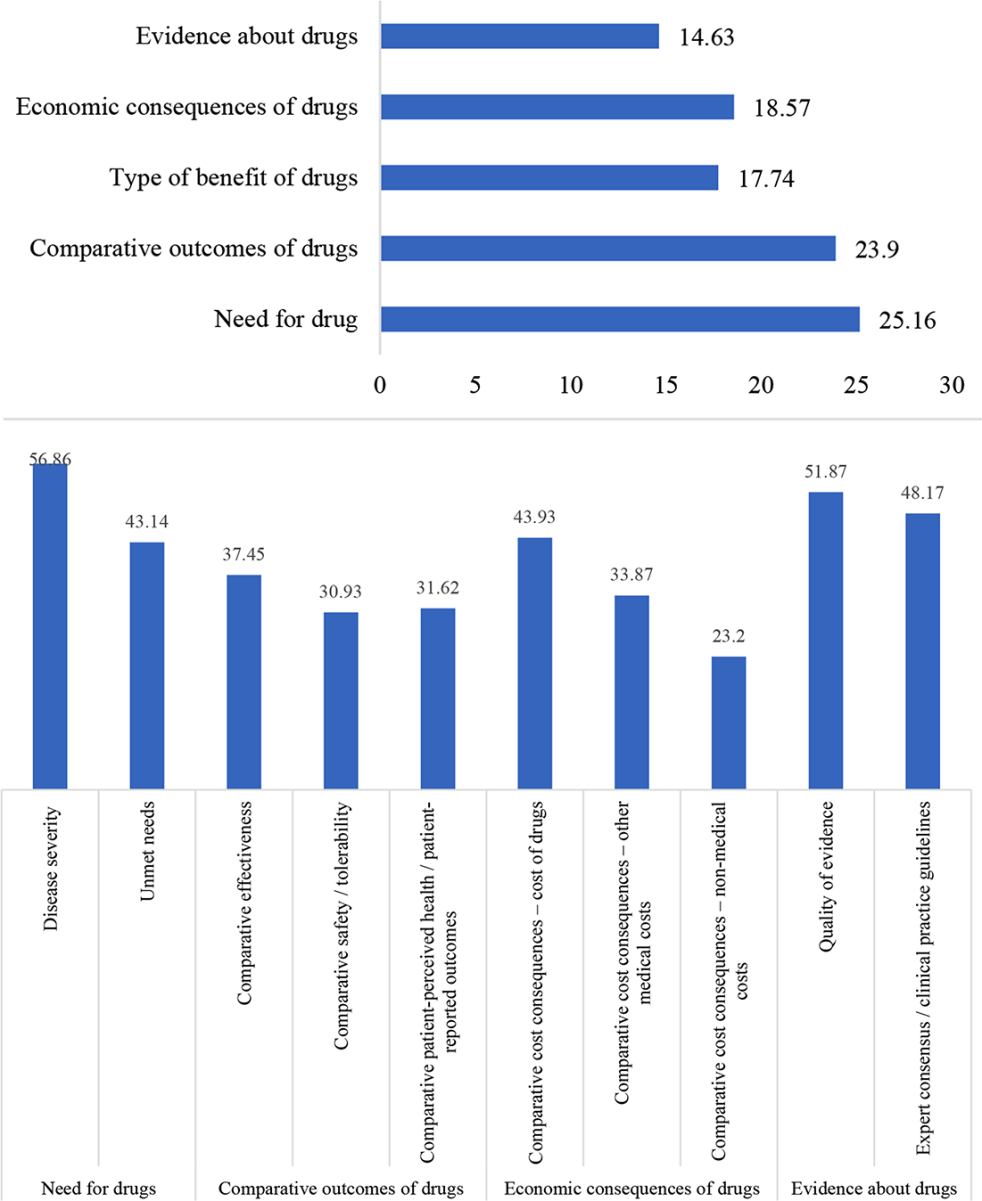
Weighting results based on two-step percentile distribution method from perspective of the public

From the perspective of the public, the weighting results measured using the two step percentile distribution methods for each criterion in the framework are shown in Fig. 3.

### Coordination analysis of the stakeholder experts’ opinions and the public

In terms of the coordination of the stakeholders’ opinions, when applying the five-point weighting method, the Kendall W of the stakeholders was greater than 0.2 (*p* < 0.05), indicating statistically significant consistency among the opinions. This reflects that different stakeholder experts had fewer suggestions regarding the weight allocation of the orphan drug value criteria. Notably, we observed that all scores of the member were greater than 0.2, and the differences in opinion among the stakeholder groups were small.

In the first-level domain of the criteria framework of the two-step percentile distribution method, the Kendall W of the stakeholders for the weight allocation of each domain was 0.37 (*p* < 0.05), indicating statistical consistency among the opinions. In addition, we also found that all scores of the member were greater than 0.2, and the differences in opinion among the stakeholder groups were small. Detailed results are provided in Appendix 10. For the second-level criteria of the criteria framework for the two-step percentile distribution method, the Kendall W was not applicable to the second weighting allocation process. Instead, we used the CV to illustrate the level of agreement among the experts for allocating weights to specific criteria. We observed that experts had different views on the importance of criteria outside of “Comparative effectiveness”, but they were consistent in their evaluation of the weight of this criterion. The details are provided in Appendix 11.

In the subgroup analysis, we found that some criteria were consistent (CV less than 0.15) within the same identity of the stakeholder as shown in Appendix 12. The CV for the opinions on different criteria ranged from 0.1 to 0.5, indicating moderate variation. However, this suggests that the allocation of weighting for orphan drug value criteria was consistent.

In terms of the coordination of the public’s opinions, the statistic results showed that the standard deviation of the weight of each criterion was 12–19, and the coefficient of variation (CV) was 0.3–0.6, indicating the preference among respondents were different, and the weight of “Type of benefit of drug” had the highest weight among the 11 criteria as shown in Appendix 13.

### Results of weighting of the value assessment criteria for orphan medicinal products from the perspective of stakeholders and the public

**Fig. 4:**
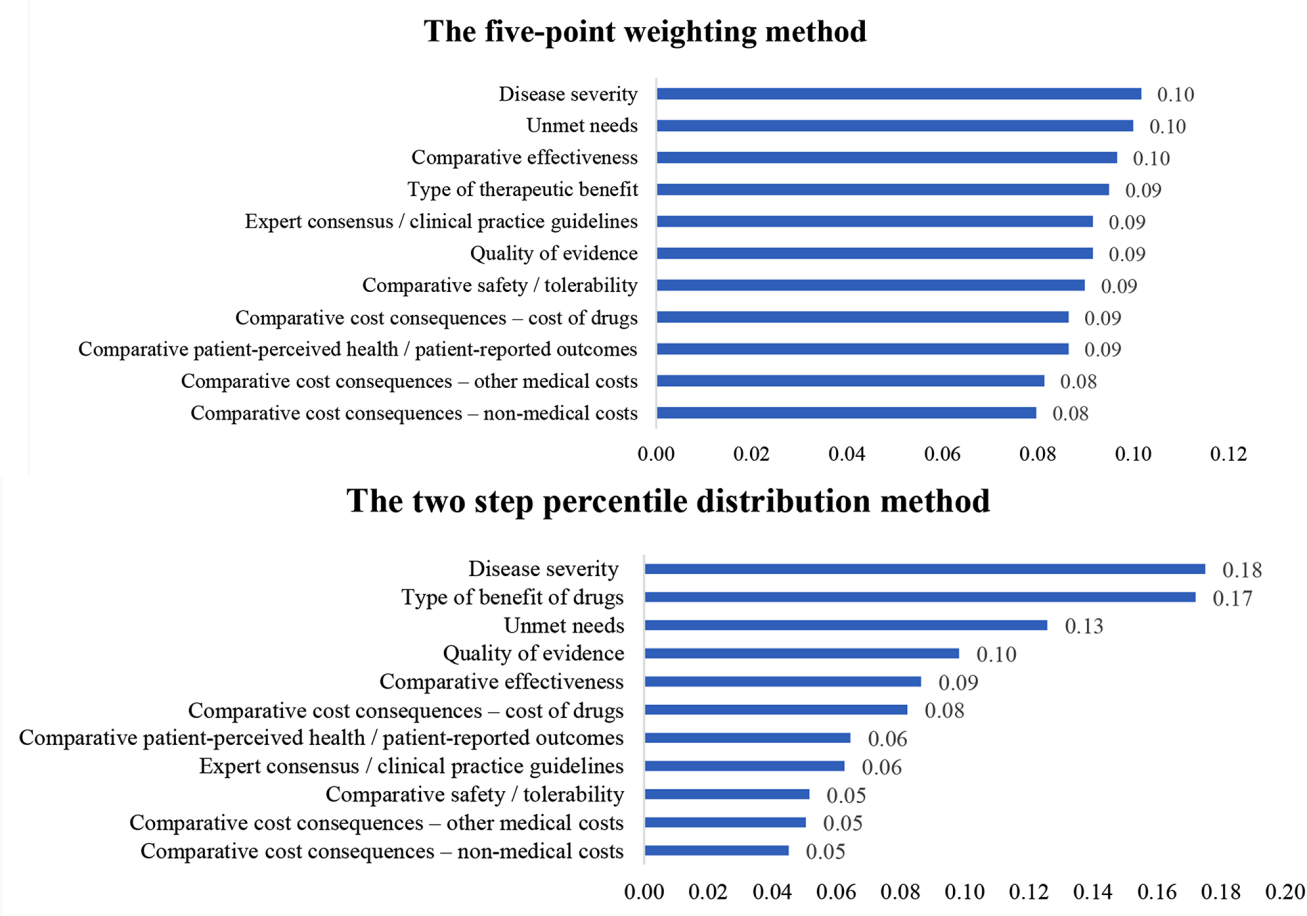
Contrast results of the five-point weighting method and the two-step percentile distribution method. Note: The total weight of all criteria was set to 1, and the individual criterion weights were normalised

The total weight of all the criteria was set to 1, and the individual criterion weights were normalised. The comparison results are detailed in Fig. 4 which indicated a large difference in weighting scores between the two methods, with the weights of each criterion being relatively close in the five-point weighting method, but quite different in the two-step percentile distribution method. Notably, the two methods had unanimous consequences in the most and least important criteria.

**Fig. 5:**
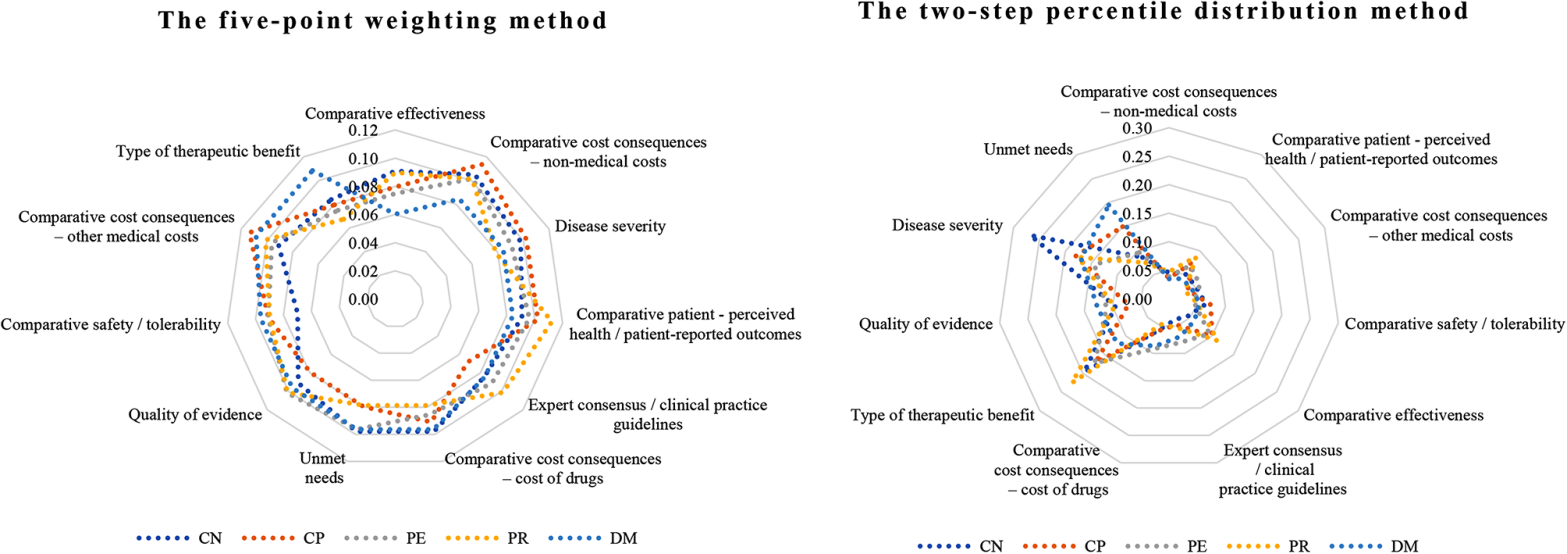
Contrast results of criteria weighting by stakeholder experts between the five-point weighting method and the two-step percentile distribution method. Notes: CN (clinicians), CP (clinician-pharmacists), DM (decision-makers), PR (patient representatives), PE (pharmaceutical economists and / or epidemiologists)

In the subgroup analysis of the weighting results of the criteria, in the five-point weighting method, the weighting allocation of the criteria between the different stakeholder groups was not significantly different. However, there was a notable difference in the order of relative importance of the criteria among different stakeholder groups. In the two-step percentile distribution method, the weighting allocation of the criteria varied between the different stakeholder groups (see Fig. 5).

From the perspective of the public, a total of 76 questionnaires on the preference of criteria were issued, with 71 valid questionnaires recovered, and the recovery rate of the valid questionnaires was 89.9%. There were significant differences among the criteria when applying the two-step percentile distribution method as well. The details are provided in Appendix 14.

## Discussions

### Progress of MCDA for value assessment of orphan medicinal products

Over the past 10 years, many studies have applied MCDA models and methods to assess the value of orphan drugs and guide medical insurance access decisions. However, most studies employ slightly different methods, have different priorities, and involve different complexities. Nevertheless, progress in the application of MCDA to practical health decisions and scientific research has been slow, and there remains no consensus on model structure, standard selection, and weighing. Of the 26 publications collected, 11 criteria selected for the framework were based on a literature review, and eight were based on the EVIDEM framework. Notably, the framework established by the literature review addresses specific decision-making issues and reflects the benefit goals of a specific population [21]. Moreover, it supports the use of discrete selection trials and other weight determination methods to better reflect the choice preferences of stakeholders and the public. However, criteria frameworks based on literature often lack maturity and have overlapping criteria. For instance, the inclusion of criteria may omit the ethical considerations, sometimes fails to satisfy the interests of all stakeholders, inclusion both clinical outcomes and cost-effectiveness as criteria in the value assessment framework and the cost-effectiveness criterion actually encompasses a part of the clinical outcomes.

The systematic literature evaluation showed that the EVIDEM framework is one of the main frameworks for MCDA in the international evaluation of drug value for rare diseases, and this framework has been used extensively in multiple countries [32–34]. Indeed, the EVIDEM framework can be employed to compare various interventions in a disease to prioritise interventions [35]. Thus, the greatest advantage of using the EVIDEM framework as opposed to the one constructed based on the literature review, is that the EVIDEM framework clearly adheres to a comprehensive set of MCDA design criteria and ethical principles and considers the interests of all stakeholders. Due to the heterogeneity of rare diseases and the lack of robust clinical evidence, making it difficult to assess using health technology assessment (HTA), MCDA is beneficial for facilitating the adjustment of the criteria framework based on the characteristics of different diseases. Additionally, MCDA can accommodate various types of evidence, including real-world evidence, expert opinions, and qualitative data, making it suitable for situations where traditional randomized controlled trials (RCTs) are scarce or infeasible. The EVIDEM framework is also practical for decision-makers due to its inclusion of both quantitative (core model) and qualitative criteria (contextual tool). We believe that conducting comparative analyses with existing frameworks, sensitivity analysis or empirical studies on specific orphan drugs using the constructed framework could identify strengths and weaknesses, as well as potential obstacles in future implementation processes. This has significant implications for developing updated and more suitable frameworks in the future.

### Value assessment framework and weighting analysis for orphan medicinal products

When inviting the panel of stakeholder experts to discuss and choose the framework criteria, they agreed that choosing the EVIDEM framework as the basis was a very good design. The EVIDEM framework follows an ethical design, and its application in the value assessment of orphan drugs is relatively mature worldwide. In addition, the stakeholder experts pointed out that when selecting criteria, it is necessary to consider the societal value of orphan drugs. For example, the pressure faced by stakeholder experts-stemming from the expectations and demands of medical institutions, government, and the public-leads to thoughtful consideration. Additionally, the disease severity draws the attention of medical institutions, government, and the public. This significantly increases the likelihood of promoting the research and innovation of orphan drugs.

In the weighting allocation, different stakeholders also showed different preferences. In the two-step percentile distribution method, when the first step allocated weights to the first-level domain of the criteria framework, the opinions of all the stakeholders were consistent. However, in the second step, when the weights were distributed between specific criteria, the opinions of all the stakeholders were not consistent. In the subgroup analysis within different stakeholders, consistent opinions were observed on the weight allocation between criteria. These results suggested that different stakeholders had varying perspectives on the value of orphan medicinal products, which could pose challenges in achieving a consensus on the weight of this study framework. Therefore, research must balance the number of stakeholders to achieve an overall balance of opinions among different stakeholders. In the aspect of the framework criteria, we also found in the studies of many countries [36, 37] such as Poland, Italy, and the Netherlands, stakeholders placed more weight on criteria related to the clinical effectiveness, safety, and evidence quality of treatments for disease severity and burden. In contrast, the final criteria framework determined using the MCDA method in this study placed more weight on disease severity, types of benefit of drugs, and unmet needs. It can be observed that stakeholders in different countries share the same views on the criteria of disease severity and quality of evidence, but there are also differing opinions on the weight of other criteria.

### Comparison results of MCDA weighting methods for orphan medicinal products

Currently, in the field of MCDA for assigning weights, the most commonly used MCDA weighting methods worldwide including the Delphi method, analytic hierarchy process (AHP), discrete choice experiment (DCE), five-point weighting method, and two-step percentile distribution method [38–42]. However, for rare diseases where stakeholders have divergent perspectives, the practical effectiveness of the Delphi method may be limited. In contrast, the AHP can transform qualitative criteria into quantitative weights [43] according to the relative importance assigned by respondents to each factor at each level. If there are too many criteria, particularly more than nine, it can lead to a time-consuming weighting workload, which may cause respondents to feel overwhelmed or confused when making judgements [44]. In contrast to the other methods discussed above, the DCE method requires a large sample size for the survey. Moreover, including an excessive number of criteria can create a significant large information burden on the survey, which can lead to poor experimental results [45]. As an example, in this study, 11 quantitative criteria were weighted using the DCE method through SAS 9.0^®^ [46], and as a result, the minimum selection set required for this method was found to be 86 choices. Compared to the two weighting methods above, this study ultimately adopted the five-point weighting method and two-step percentage distribution method for weighting, which has been applied in many studies [41, 42]. They belong to the direct scoring method, support the weighting of a larger number of criteria, and are simpler to operate.

Likewise, regarding the consistency of the stakeholders’ weight allocations, the five-point weighting method was more consistent. Some studies have shown that the suggestions from stakeholders were mainly supportive, and moderate choices were often made when using simple scoring methods [47]. The results of this study reflected this problem, and in the five-point weighting method, stakeholders tended to assign four or five points to all criteria, which did not truly reflect the stakeholders’ preferences for different value criteria for rare diseases. However, in the two-step percentile distribution method, the differences between the criteria were more evident. We also found that the two-step percentile distribution method inherently encouraged more active participation from stakeholders. The first step of assigning weights to criteria allows stakeholders to express their priorities quantitatively, while the second step refines these preferences through discussion and consensus-building. This method ensures that stakeholders are not just contributors but active participants in the decision-making process, leading to more comprehensive and representative outcomes. Furthermore, the two-step percentile distribution is particularly able to capture the breadth of stakeholder perspectives because it allows for the nuanced expression of preferences. Stakeholders can assign precise percentages to reflect the importance they attribute to each criterion, accommodate a wide range of views. Consequently, the two-step percentile distribution method is better than the five-point weighting method, as the latter may have too balanced weight allocation and cannot distinguish heterogeneity among different criteria. It can better reflect the different views of different stakeholder experts on the value criteria of orphan medicinal products and elicit more positive feedback from interested experts, resulting in significantly different weight allocations among criteria.

### Limitations

Due to the COVID-19 pandemic, this study was designed to be conducted in two workshops via an online conference platform. Compared to on-site discussions, it is more difficult for stakeholders to reach a consensus through face-to-face and in-depth discussions, resulting in insufficient consistency of results. In future studies, we propose that implementing scenario or sensitivity analysis could more effectively address the complexities and uncertainties involved in evaluating the value of orphan drugs during a pandemic. In addition, the number of stakeholders included in this study was not balanced, and some clinical pharmacy experts were pharmacoeconomic experts, resulting in a higher proportion of pharmaceutical economist experts, which may have caused bias in the results. Therefore, for future occurrences, we recommend that conducting in-depth questionnaires or face-to-face conversations with stakeholders before their inclusion, which will help to understand their potential professional backgrounds better and prevent scenarios where a stakeholder might represent multiple different backgrounds. Additionally, to ensure more equitable participation among different stakeholders, we could focus on balancing the number ratio of different stakeholders to guarantee more equitable involvement from various parties in future research. To broaden participation, we believe that we could hold open meetings to facilitate discussions among a wider audience. Furthermore, this study aimed to construct a framework for assessing the value of orphan medicinal products without including criteria for specific diseases or access situations. Therefore, the relevance and applicability of the criteria may be limited when assessing specific drugs in certain disease contexts or access situations.

## Conclusion

Based on the literature and the EVIDEM framework, this study drafted an initial value assessment framework. Through two stakeholder expert workshops, the criteria and weights for the value assessment framework of orphan medicinal products were established. Following this, the entire framework of the value assessment criteria of orphan drugs based on MCDA was preliminarily established. Unlike the traditional “cost-effectiveness” drug value assessment model, this study constructed a criteria framework that considers the societal value attributes of orphan drugs and focuses on the value preferences of the stakeholders themselves. Thus, in the value assessment and decision dilemma, such a value assessment framework with broader societal value criteria is more conducive to assisting decision making.

## Electronic supplementary material

Below is the link to the electronic supplementary material.

Supplementary Material 1

## Data Availability

All data are publicly available.

## Abbreviations

AHP: Analytic hierarchy process
DCE: Discrete choice experiment
EVIDEM: Evidence and Value Impact on Decision Making
HTA: Health technology assessment
MCDA: Multi-criteria decision analysis
OMP: Orphan medicinal products
RCT: Randomized controlled trials
SPIDER: Sample, Phenomenon of Interest, Design, Evaluation, Research type
