# Supplementary material for: Establishment of a value assessment framework for orphan medicinal products in China
Source: Orphanet J Rare Dis. 2024 Oct 20;19:390. doi: 10.1186/s13023-024-03393-6 (PMC11492536; doi:10.1186/s13023-024-03393-6)

**Establishment of a value assessment framework for orphan medicinal products in China (Appendices 1-14)**

**Appendix 1. The initial version of the value assessment framework for orphan medicinal product**

| **Domain / Criteria** | **Possible Sub-criteria** | **Definitions** | Should be Considered?  Delete this column if criteria selection / validation is not part of the workshop |
| --- | --- | --- | --- |
| **EVIDEM core model (criteria appraised quantitatively)** | | | |
| **Need for drugs** | | | |
| Disease severity | - Effect of disease on life-expectancy - Effect of disease on morbidity (includes disability and function) - Effect of disease on patient’s quality of life - Effect of disease on caregivers’ quality of life | Severity of the health condition of patients treated with the proposed drug (or severity of the health condition that is to be prevented) with respect to mortality, morbidity, disability, function, impact on quality of life, clinical course (i.e., acuteness, clinical stages). | Yes 🞎  No 🞎 |
| **Size of affected population** | - Prevalence - Incidence | Number of people affected by the condition (treated or prevented by the proposed drug) among a specified population at a specified time; can be expressed as annual number of new cases (annual incidence) and/or proportion of the population affected at a certain point in time (prevalence). | Yes 🞎  No 🞎 |
| **Unmet needs** | - Unmet needs in efficacy - Unmet needs in safety - Unmet needs in patient-reported outcomes - Patient demand | Shortcomings of comparative drugs in their ability to prevent, cure, or ameliorate the safety, patient-reported outcomes and convenience. | Yes 🞎  No 🞎 |
| **Comparative outcomes of drugs** | | | |
| **Comparative effectiveness** | - Magnitude of health gain - Percentage of the target population expected to realize the anticipated health gain - Onset and duration of health gain - Sub-criteria for the measure of efficacy specific the therapeutic area | Capacity of the drug to prevent or to produce a desired (beneficial) change in signs, symptoms, or course of the targeted condition above and beyond beneficial changes produced by alternative drugs. | Yes 🞎  No 🞎 |
| **Comparative safety / tolerability** | - Adverse events - Serious adverse events - Fatal adverse events - Short-term safety - Long-term safety - Tolerability | Capacity of the proposed drug to produce a reduction in drug-related harmful or undesired health effects compared to alternative drugs. | Yes 🞎  No 🞎 |
| **Comparative patient-perceived health / patient-reported outcomes** | - Improvement in health-related quality of life - Impact on autonomy - Impact on dignity - Convenience / ease of use / mode & setting of administration | Capacity of the drug to produce beneficial changes inpatient-perceived health and patient-reported outcomes (PROs) (e.g., quality of life) above and beyond beneficial changes produced by alternative drugs; also includes improvement in convenience to patients. | Yes 🞎  No 🞎 |
| **Type of benefit of drugs** | | | |
| **Type of preventive benefit** |  | Nature of the preventive benefit or risk reduction provided by the proposed drug at the population-level (e.g., eradication, prevention, reduction in disease transmission, reduction in the prevalence of risk factors). Public health perspective. | Yes 🞎  No 🞎 |
| **Type of therapeutic benefit** |  | Nature of the clinical benefit provided by the drug at the patient level (e.g., symptom relief, prolonging life, cure). | Yes 🞎  No 🞎 |
| **Economic consequences of drugs** | | | |
| **Comparative cost consequence - cost of drug** | - Net cost of drug - Acquisition cost - Implementation / maintenance cost | Net cost of covering the drug (excluding other spending). This represents the differential between expected expenditure for the drug and potential cost savings that may result from replacement of other drugs currently covered by the health plan. Limited to cost of drug (e.g. acquisition cost, implementation and maintenance cost).  Note: in countries where part of the drug cost is paid by patients (e.g. copayment), this criterion should be adjusted ~~consequently.~~ | Yes 🞎  No 🞎 |
| **Comparative cost consequences - other medical costs** | - Impact on primary care expenditures - Impact on hospital care expenditures - Impact on long-term care expenditures | Impact of the proposed drug on other medical costs (excluding drug cost) such as hospitalization, specialist consultations, adverse events costs, long-term care, etc. | Yes 🞎  No 🞎 |
| **Comparative cost consequences - non-medical costs** | - Impact on productivity - Financial impact on patients - Financial impact on caregivers - Cost to the wider social care system | Impact of the proposed drug on non-medical costs (excluding drug cost) such as disability costs, social services, lost productivity, caregiver time, etc. | Yes 🞎  No 🞎 |
| **Knowledge about drugs** | | | |
| **Quality of evidence** | - Validity - Relevance - Completeness of reporting - Type of evidence | Extent to which evidence on the drug is relevant to the decision-making body (in terms of population, disease stage, comparator drugs, outcomes, etc.) and valid with respect to scientific standards (i.e., study design, etc.) and conclusions (i.e., agreement of results between studies). This includes consideration of uncertainty (e.g., conflicting results across studies, limited number of studies and patients). Complete reporting of evidence is a pre-requisite to assess coherence and validity. | Yes 🞎  No 🞎 |
| **Expert consensus / clinical practice guidelines** |  | Concurrence of the drug (similar alternatives) with the current consensus of experts on what constitutes state-of-the-art practices in the management of the targeted health condition; clinical practice guidelines are usually developed via an explicit process that combines formal and expert knowledge, with the intent to improve clinical practice. | Yes 🞎  No 🞎 |
| **EVIDEM contextual tool (criteria appraised qualitatively)** | | | |
| **Normative contextual criteria** | | | |
| **Mandate and scope of healthcare system** |  | Alignment of the drug with the mandate / scope of the healthcare system. The goal of healthcare is to maintain normal functioning. Mission and scope of healthcare plans/systems derive from this principle. | Yes 🞎  No 🞎 |
| **Population priorities and access** | - Current priorities of health system (e.g., disabled; low socioeconomic status; specific age groups) - Special populations (e.g., ethnicity) - Remote communities - Rare diseases - Specific therapeutic areas | Alignment of the drug with current priorities of health  System / plan. Priorities for specific groups of patients are defined by societies / decision makers and reflect their moral values. Such considerations are aligned with the principle of justice, which considers treating like cases alike and different cases differently and often gives priority to those who are worst-off. | Yes 🞎  -Add to quantitative appraisal 🞎  No 🞎 |
| **Common goal and specific interests** | - Stakeholders’ pressures - Stakeholders’ barriers - Conflict of interest | Pressures or barriers from groups of stakeholders or individuals are often part of the context surrounding healthcare drugs. Being aware of pressures and interests at stake and how they may affect decision making helps ensure that decisions are aligned with the common goal. | Yes 🞎  No 🞎 |
| **Environmental impact** | - Environmental impact of production - Environmental impact of use - Environmental impact of implementation | The extent to which the production, use or implementation of the drug causes environmental damages. | Yes 🞎  -Add to quantitative appraisal 🞎  No 🞎 |
| **Feasibility contextual criteria** | | | |
| **System capacity and appropriate use of drug** | - Organizational requirements (e.g., process, premises, equipment) - Skill requirements - Legislative requirements - Surveillance requirements - Risk of inappropriate use - Institutional limitations to uptake - Ability to reach the whole target region/population | The capacity of a healthcare system to implement the drug and to ensure its appropriate use depends on its infrastructure, organization, skills, legislation, barriers, and risks of inappropriate use. Such considerations include mapping current systems and estimating whether the use of the drug under scrutiny requires additional capacities. | Yes 🞎  -Add to quantitative appraisal 🞎  No 🞎 |
| **Political / historical / cultural context** | - Political priorities and context - Cultural acceptability - Precedence (congruence with previous and future decisions) - Impact on innovation & research - Impact on partnership & collaboration among healthcare stakeholders | The political, historical, or cultural context may influence the value of a drug with respect to specific political situations and overall priorities (e.g., priority for innovation) as well as habits, traditions, and precedence. | Yes 🞎  No 🞎 |
| **Opportunity cost** | | | |
| **Opportunity costs and affordability** | - Opportunity costs for patient (forgone resources) - Opportunity costs for population (forgone resources) - Affordability | Consideration of the medical resources that may be forgone  (opportunity costs) if the drug is implemented and whether the healthcare system can afford implementing the drug. Both affordability and opportunity cost considerations require a financial / budgeting exercise. Opportunity costs and affordability can be considered at the system/institution level and at the patient level. | Yes 🞎  No 🞎 |
| **Other criteria to consider? 1. ________________; 2. ________________** | | | |

**Appendix 2. Detailed information with regarding to the stakeholder experts within the panel.**

| **NO** | **Institute** | **Professional** | **Types of the stakeholder member** |
| --- | --- | --- | --- |
| 1 | Home of Patient with Multiple Sclerosis in China | Founder | PR |
| 2 | The Illness Challenge Foundation | Chief Medical Officer, CMO | PR |
| 3 | Shanghai Rare Disease Prevention and Treatment Foundation | President | CN |
| 4 | West China Hospital, Sichuan University | Chief Physician | CN |
| 5 | Guangzhou Women and Childrens Medical Center | Chief Physician | CN |
| 6 | Department of Pharmacy, Peking Union Medical College Hospital | Associate Chief Pharmacist | CP |
| 7 | Department of Pharmacy, Renji Hospital, Shanghai Jiao Tong University School of Medicine | Associate Chief Pharmacist | CP |
| 8 | Fudan University | Professor | PE |
| 9 | Tianjin University | Professor | PE |
| 10 | Peking University | Professor | PE |
| 11 | Sichuan University | Professor | PE |
| 12 | Shanghai Health and Health Development Research Center | Associate Researcher | PE |
| 13 | Beijing Friendship Hospital, Capital Medical University | Director of the Healthcare Security Department | DM |
| 14 | Beijing Society for Rare Disease Diagnosis, Treatment and Protection | Vice-Chairman | DM |
| 15 | Sichuan Provincial Healthcare Security Administration | Minister | DM |

**Notes:** CN(clinicians), CP(clinician pharmacists),DM(decision maker), PR(patient representative), PE(Pharmaceutical economists and epidemiologists)

**Appendix 3. Inclusion of the expert panel of stakeholders**

| Types of stakeholder members | | Inclusion guidelines of the stakeholder experts | Number |
| --- | --- | --- | --- |
| PR | ·Active members of the local patient support group in the rare disease field. | 2 |  |
| CN | ·Active practitioners engaged in professionally treating patients with rare diseases.  ·Practitioners working with prestigious rare diseases.  ·Authors of famous guidelines or other publications in the field of rare diseases.  ·Professionals with no conflicts of interest. | 3 |  |
| PE | ·Academic / consulting professionals within an active research team related to the decision-making field. | 5 |  |
| CP | ·Professionals working in the prestigious rare disease field.  ·Authors of famous guidelines or other publications in the field of rare diseases.  · Professionals with no conflicts of interest. | 2 |  |
| CM | ·Members of relevant HTA decision-making or recommendation development agencies.  ·Ideally, professionals involved in decision-making processes. | 3 |  |

**Notes:** CN (clinicians), CP (clinician-pharmacists), DM (decision-makers), PR (patient representatives), PE (pharmaceutical economists and/or epidemiologists), HTA (health technology assessment)

**Appendix 4. The questionnaires of weighting for orphan drugs based on MCDA**

**Appendix 4.1 The five-point weighting method**

Note: Please scan the QR code and open Questionnaire 1, then we ask the stakeholder experts to review one by one, from their own point of view, according to its relative importance of each criterion assigned 1-5 points of weight.

**Questionnaire 1:**

| **Weighting method: the five-point weighting method** | |
| --- | --- |
| **Instructions：**  From your perspective, assign a weight to each criterion of decision according to its relative importance when appraising a healthcare drug in the context of appraisal  Assign 5 to the criteria you consider most important  Assign 1 to the criteria you consider least important  Note: The part of the questionnaire should be filled out online and applicable weights will be normalized. | |
| Domains / Criteria | Weight (relative importance)  low high |
| Need for drugs | |
| Disease severity  What is the relative importance of the severity of the disease targeted by the drug? | 1  2  3  4  5 |
| Size of affected population  What is the relative importance of the size of the population targeted by the drug? | 1  2  3  4  5 |
| Unmet needs  What is the relative importance of the level of unmet needs to manage this disease? | 1  2  3  4  5 |
| Comparative outcomes of drugs | |
| Comparative effectiveness  What is the relative importance of the efficacy / effectiveness of the drug compared to its alternatives? | 1  2  3  4  5 |
| Comparative safety / tolerability  What is the relative importance of the safety of the drug compared to its alternatives? | 1  2  3  4  5 |
| Comparative patient-perceived health / patient-reported outcomes  What is the relative importance of the patient-perceived health / patient-reported outcomes of the drug compared to its alternative? | 1  2  3  4  5 |
| Type of benefit of drugs | |
| Type of preventive benefit  What is the relative importance of the type of preventative health benefit (e.g., risk reduction) provided by the drug. | 1  2  3  4  5 |
| Type of therapeutic benefit  What is the relative importance of the type of therapeutic health benefit provided by the drug. | 1  2  3  4  5 |
| Economic consequences of drugs | |
| Comparative cost consequences - cost of drug  What is the relative importance of the direct cost impact of the drug (including acquisition, implementation and maintenance costs)? | 1  2  3  4  5 |
| Comparative cost consequences - other medical costs  What is the relative importance of the impact of the drug on other medical costs such as hospitalization, specialist consultations, adverse events costs, long-term care, etc.? | 1  2  3  4  5 |
| Comparative cost consequences - non- medical costs  What is the relative importance of the impact of the drug on non-medical costs such as disability costs, social services, lost productivity, caregiver time, etc.? | 1  2  3  4  5 |
| Evidence about drugs | |
| Quality of evidence  What is the relative importance of the quality of the design of studies and their relevance to the context? | 1  2  3  4  5 |
| Expert consensus / clinical practice guidelines  What is the relative importance of the recommendations on product (or products of the same class) in well-established guidelines? | 1  2  3  4  5 |
| Other domains / criteria  Note: Adapt MCDA Core Model to specific applications by adding domains / criteria / sub-criteria (see above and see also details on adaptation available in the EVIDEM v4.0 Concepts & definitions) | |
|  | 1  2  3  4  5 |
|  | 1  2  3  4  5 |

**Appendix 4.2 The two-step percentile distribution method**

Note: Please scan the QR code and open the questionnaire 2, then we ask the stakeholder experts to review one by one, from their own point of view, according to its relative importance of each criterion assigned 100 points of weight to the first level domain and then assigned 100 points of weight between the second-level criteria under each domain.

**Questionnaire 2:**

| **Weighting method: the two-step percentile distribution method** |
| --- |
| **Instructions：**  Assigning scores to domain / criteria based on how much weights (relative importance) you think should be given to a particular domain / criterion.  Assign 100 points of weight to the first level domain;  Assign 100 points of weight to the second level criteria under each domain;  Note: The part of the questionnaire 2 should be filled out online. |


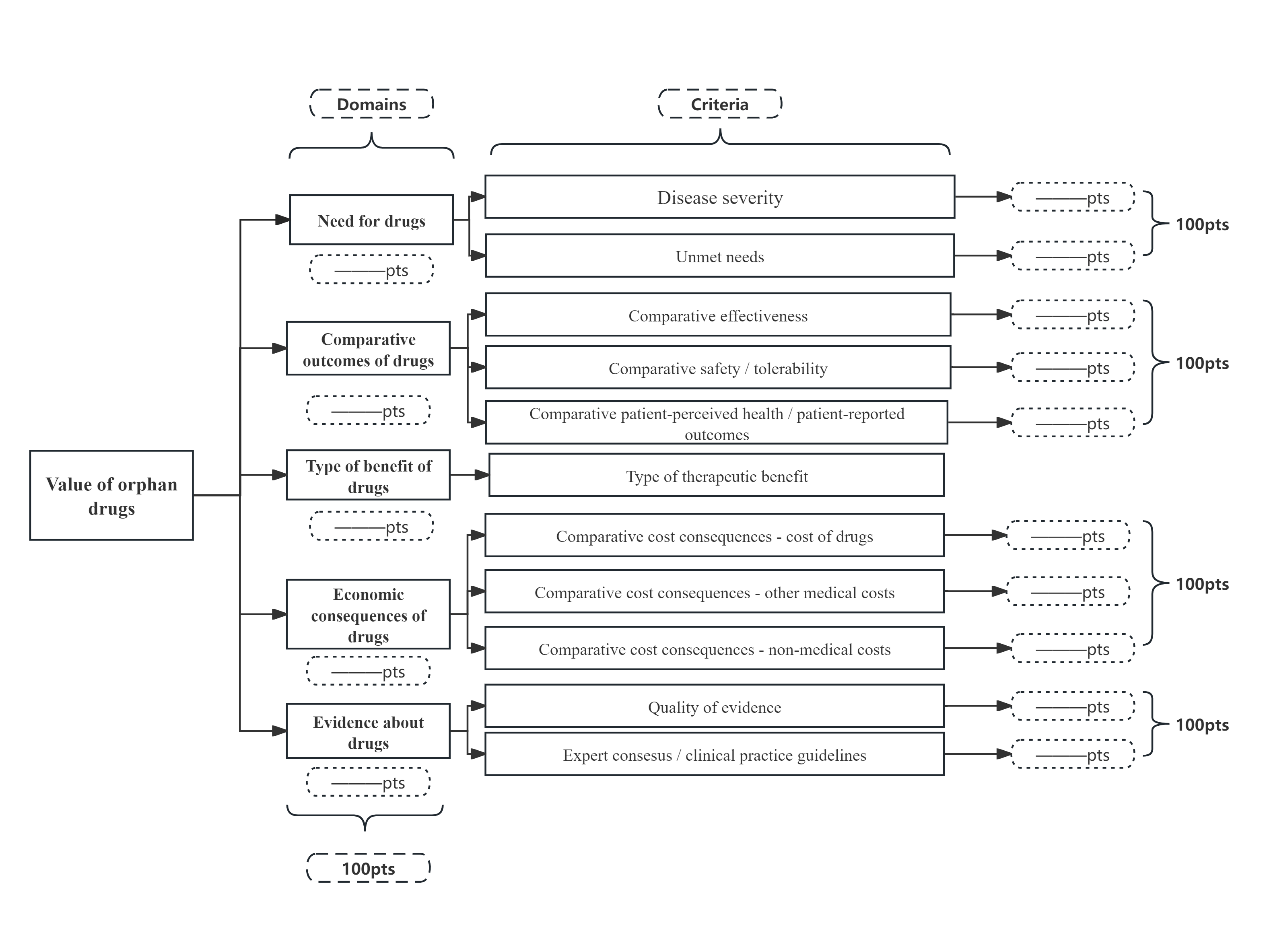


**Appendix 5. Quota standard table of questionnaire**

| **Demographic characteristics** | **Portion of**  **China's adult population** | **Quota sample size N=60** |
| --- | --- | --- |
| **Sex** |  |  |
| male | 51.24% | 31 |
| female | 48.76% | 29 |
| **Age** |  |  |
| 18-29 | 14.84% | 9 |
| 30-39 | 16.97% | 10 |
| 40-49 | 15.75% | 9 |
| 50-59 | 16.92% | 10 |
| ≥60 | 35.52% | 21 |
| **Educational level** |  |  |
| Primary school or below | 35.08% | 21 |
| Junior high school | 38.98% | 23 |
| High school | 15.85% | 10 |
| University or above | 10.09% | 6 |
| **Household registration type** |  |  |
| Urban | 63.89% | 38 |
| Rural | 36.11% | 22 |

**Appendix 6. Quantitative criteria selection vote results in the core model of value assessment framework for orphan medicinal product based on MCDA**

| **Name of criteria** | **Criteria selection vote results**  **(Consent / total)** |
| --- | --- |
| **Quantitative criteria of the value assessment framework (core model)** | |
| **Need for drugs** | |
| Disease severity | 10/10 |
| Size of affected population | 8/10 |
| Unmet needs | 10/10 |
| **Comparative outcomes of drugs** | |
| Comparative effectiveness | 10/10 |
| Comparative safety / tolerability | 9/10 |
| Comparative patient-perceived health / patient-reported outcomes | 9/10 |
| **Type of benefit of drugs** | |
| Type of preventive benefit | 7/10 |
| Type of therapeutic benefit | 10/10 |
| **Economic consequences of drugs** | |
| Comparative cost consequences – cost of drugs | 9/10 |
| Comparative cost consequences – other medical costs | 8/10 |
| Comparative cost consequences – non-medical costs | 6/10 |
| **Evidence about drugs** | |
| Quality of evidence | 9/10 |
| Expert consensus / clinical practice guidelines | 8/10 |

**Appendix 7. Qualitative criteria selection vote results in the contextual tools of value assessment framework for orphan medicinal product based on MCDA**

| Name of criteria | **Criteria selection vote results**  (Consent / total) | Add to quantitative appraisal  (Consent / total) |
| --- | --- | --- |
| Qualitative criteria of the value assessment framework（contextual tool) | | |
| Normative contextual criteria | | |
| Mandate and scope of healthcare system | 8/10 |  |
| Population priorities & access | 9/10 | 4/10 |
| Common goal and specific interests | 7/10 |  |
| Environmental impact | 5/10 | 3/10 |
| Feasibility contextual criteria | | |
| System capacity and appropriate use of the drug | 9/10 | 6/10 |
| Political / historical / cultural context | 4/10 |  |
| Opportunity costs & affordability | 10/10 |  |
| Other criteria added in the workshop | | |
| Medicare accessibility | 3/3 |  |
| Drug multiple indications | 2/2 |  |

**Appendix 8. Expert opinions and criteria modifications in the first workshop for orphan drug value assessment based on MCDA**

| **Number** | **Stakeholder experts’ opinions and suggestions** | **Revised criteria lists, as recommended** |
| --- | --- | --- |
| 1 | It is recommended to remove the criterion “Size of affected population”, as this framework has been defined for orphan drug value assessment and the population size of those with rare diseases is very small; thus, this criterion is not required. (DM1, DM2, CP2, CN2) | The criterion “Size of affected population” was removed. (CN1) |
| 2 | It is recommended to consider the value of orphan drug dose types in the criterion “Comparative patient-perceived health / patient-reported outcomes”. (PR2) | The sub-criterion “Dosage form” was added to the criterion “Comparative patient-perceived health / patient reported outcomes”. |
| 3 | It is recommended to remove the criterion “Type of preventive benefit”, as the vast majority of orphan drugs have no function in the public health perspective. (CN1, CN2, CP2) | The criterion “Size of affected population” was removed. (CN3) |
| 4 | It is recommended to consider adding criteria to determine whether the orphan drugs can be accessible and prescribed in the designated medical institutions of the Rare Disease Collaborative Network. (CN3, DM1) | The “Institutional requirements” criterion, a sub-criterion of “System adaptability and rational use of drugs”, was changed to “requirements for designated medical institutions of the Rare Disease Cooperative Network”. (CP4) |
| 5 | It is suggested that a drug should have added value if it fits within an established policy. (PR2) | The qualitative criterion “Government objectives and policy priorities” was added. (CP5) |
| 6 | It is recommended to consider whether the orphan drug is covered by medical aid and whether the aid will be cancelled if the drug is covered by medical insurance. (CP2) | The qualitative criterion “Aid program sustainability” was added. (PE6) |
| 7 | It is suggested that this research framework should consider the criteria for scientific and technological innovation, as scientific and technological innovation will encourage drug companies to develop drugs for rare diseases. (CN 3, PR2) | The qualitative criterion “Technological innovation” was added. (PE7) |
| 8 | It is suggested to consider the budget impact and the affordability of medical insurance funds. (CP1, DM1) | The qualitative criterion “Opportunity costs and affordability” was changed to “Affordability of medical insurance funds”. |
| 9 | Sixty percent of the experts did not choose to include the qualitative criterion “political, historical, and cultural context” in the framework. | The qualitative criterion “Political / historical / cultural context” was removed (according to criteria selection vote results by the stakeholders). |

**Notes:** CN (clinicians), CP (clinician-pharmacists), DM (decision-makers), PR (patient representatives), PE (pharmaceutical economists and/or epidemiologists)

**Appendix 9. Results of the online questionnaire by the stakeholder experts**

| Domains / criteria | Consent rate for inclusion models  (consent / total) | Number of quantitative models included | Rate of scaling  (consent / total) |
| --- | --- | --- | --- |
| Quantitative criteria of the value assessment framework (core model) | | | |
| Need for drugs | | | |
| Disease severity | 13/13 | - | 15/15 |
| Unmet needs | 13/13 | - | 15/15 |
| Comparative outcomes of the drugs | | | |
| Comparative effectiveness | 13/13 | - | 15/15 |
| Comparative safety / tolerability | 13/13 | - | 15/15 |
| Comparative patient-perceived health / patient-reported outcomes | 13/13 | - | 15/15 |
| Benefit types of the drugs | | | |
| Types of therapeutic benefits | 12/13 | - | 15/15 |
| Economic consequences of the drugs | | | |
| Comparative cost consequences – cost of drugs | 9/13 | - | 13/15 |
| Comparative cost consequences – other medical costs | 10/13 | - | 13/15 |
| Comparative cost consequences – non-medical costs | 8/13 | - | 13/15 |
| Knowledge about the drugs | | | |
| Quality of evidence | 11/13 | - | 15/15 |
| Expert consensus / clinical practice guidelines | 8/13 | - | 13/15 |
| Qualitative criteria of the value assessment framework (Contextual tool) | | | |
| Normative contextual criteria | | | |
| Mandate and scope of the healthcare system | 12/13 | - | 12/15 |
| Population priorities and access | 11/13 | 6/13 | 13/15 |
| Common goal and specific interests | 9/13 | - | 12/15 |
| Feasibility contextual criteria | | | |
| Environmental impact | 6/13 | 3/13 | 13/15 |
| System capacity and appropriate use of the drug | 9/13 | 4/13 | 13/15 |
| Government objectives and policy priorities | 11/13 | 6/13 | 14/15 |
| Aid program sustainability | 8/13 | 3/13 | 13/15 |
| Technological innovation | 10/13 | 6/13 | 13/15 |
| Affordability of medical insurance funds | 11/13 | 9/13 | 13/15 |

**Note:** Thirteen and 15 stakeholder experts participated in the process of criteria rectification and criteria scoring scale creation, respectively. The process of criteria rectifying included 3 clinicians, 2 clinical pharmacists, 3 pharmaceutical economists and/or epidemiological experts, 2 patient representatives, and 3 decision-makers. The criteria scoring scale creation process included 3 clinicians, 2 clinical pharmacists, 6 pharmaceutical economists and/or epidemiological experts, 2 patient representatives, and 3 decision-makers.

According to the results of the online questionnaire from the stakeholder experts, the framework removed the criterion "environmental impact". Among the quantitative criteria, stakeholder experts suggested that "affordability of medical insurance funds" should be included. However, contextual tools are also important for decision making. The criteria in the contextual tool are all qualitative and do not have weights assigned to them. This prioritises the top-ranked orphan drugs through the core model. Additionally, a qualitative evaluation using the contextual tool was performed for the collected empirical evidence on orphan drugs. This can help decision-makers make the final judgement on whether to include orphan drugs in medical insurance. In addition, the affordability of medical insurance funds is very important when included in national medical insurance decisions; therefore, this was not included in the quantitative criteria.

**Appendix 10. Coordination results of stakeholder experts weighting allocation**

| Types of the stakeholder member | Number | Coordination coefficient within the expert group (the five-point weighting method) | Coordination coefficient within the expert group (the two-step percentile distribution method) |
| --- | --- | --- | --- |
| CN | 3 | 0.49 | 0.47 |
| CP | 2 | 0.59 | 0.8 |
| PE | 4 | 0.46 | 0.32 |
| PR | 2 | 0.67 | 0.74 |
| DM | 2 | 0.27 | 0.61 |
| the Kendall coordination coefficient(W) | | 0.21 | 0.37 |
| The asymptotic significance | | 0.002 | 0.002 |

**Notes:** CN(clinicians), CP(clinician pharmacists),DM(decision maker), PR(patient representative), PE(Pharmaceutical economists and epidemiologists).

**Appendix 11. Coordination results between criteria within the second level of stakeholder experts in two-step percentile distribution method**

| Types of the member | Number | Coefficient of variation within the stakeholders' expert group | | | | | | | | | | |
| --- | --- | --- | --- | --- | --- | --- | --- | --- | --- | --- | --- | --- |
|  |  | **Need for drugs** | | **Comparative outcomes of drugs** | | | **Type of benefit of drugs** | **Economic consequence of drugs** | | | **Knowledge about drugs** | |
|  |  | Disease severity | Unmet needs | Comparative effectiveness | Comparative safety / tolerability | Comparative patient-perceived health / patient-reported outcomes | Type of therapeutic benefit | Comparative cost consequences – cost of drugs | Comparative cost consequences – other medical costs | Comparative cost consequences – non-medical costs | Quality of evidence | Expert consensus / clinical practice guidelines |
| CN | 3 | 0.24 | 0.74 | 0.76 | 0.39 | 0.18 | 0.42 | 0.08 | 0.11 | 0.15 | 0.12 | 0.29 |
| CP | 2 | 0.10 | 0.12 | 0.00 | 0.36 | 0.34 | 0.06 | 0.06 | 0.25 | 0.16 | 0.33 | 0.50 |
| PE | 4 | 0.22 | 0.34 | 0.17 | 0.40 | 0.13 | 0.26 | 0.21 | 0.09 | 0.42 | 0.16 | 0.23 |
| PR | 2 | 0.14 | 0.33 | 0.11 | 0.00 | 0.14 | 0.33 | 0.60 | 0.20 | 0.47 | 0.22 | 0.41 |
| DM | 2 | 0.29 | 0.25 | 0.05 | 0.14 | 0.11 | 0.17 | 0.24 | 0.13 | 0.43 | 0.05 | 0.10 |
| Coefficient of Variation  (CV) | | 0.24 | 0.34 | 0.15 | 0.37 | 0.21 | 0.37 | 0.31 | 0.18 | 0.49 | 0.21 | 0.33 |

**Notes:** CN(clinicians), CP(clinician pharmacists),DM(decision maker), PR(patient representative), PE(Pharmaceutical economists and epidemiologists)

**Appendix 12. Consistent criteria (CV less than 0.15) within same identity of the stakeholder**

| **Types of the member** | **Consistent criteria** |
| --- | --- |
| CN | “Comparative cost consequences - cost of drugs”, “Comparative cost consequences - other medical costs”, “Comparative cost consequences - non-medical costs”, and “Quality of evidence”. |
| CP | “Disease severity”, “Unmet need”, “Comparative effectiveness”, “Type of therapeutic benefit”, and “Comparative cost consequences – cost of drugs”. |
| PE | “Comparative patient-perceived health / patient-reported outcomes” and “Comparative cost consequences – other medical costs”. |
| PR | “Disease severity”, “Comparative effectiveness”, “Comparative safety / tolerability”, and “Comparative patient-perceived health / patient-reported outcomes”. |
| DM | “Comparative effectiveness”, “Comparative safety / tolerability”, “Comparative patient-perceived health / patient-reported outcomes”, “Comparative cost consequences – other medical costs”, “Quality of evidence”, and “Expert consensus / clinical practice criteria”. |

**Notes:** CN(clinicians), CP(clinician pharmacists),DM(decision maker), PR(patient representative), PE(Pharmaceutical economists and epidemiologists)

**Appendix 13. Importance preference of criteria of value assessment framework for orphan medicinal products**

| **Domain** | **Preference Score**  **of domian** | **Criteria** | **Preference Score**  **of criteria** | **CV** | **Weight of the criteria** |
| --- | --- | --- | --- | --- | --- |
| Need for drugs | 25.16±12.89 | Disease severity | 56.86±18.85 | 0.33 | 14.31% |
|  |  | Unmet needs | 43.14±18.85 | 0.44 | 10.86% |
| Comparative outcomes of drugs | 23.90±9.61 | Comparative effectiveness | 37.45±15.24 | 0.41 | 8.95% |
|  |  | Comparative safety / tolerability | 30.93±12.83 | 0.41 | 7.39% |
|  |  | Comparative patient - perceived health / patient-reported outcomes | 31.62±16.46 | 0.52 | 7.56% |
| Type of benefit of drugs | 17.74±9.90 | type of therapeutic benefit |  | 0.56 | 17.74% |
| Economic consequences of drugs | 18.57±9.84 | Comparative cost consequences – cost of drugs | 43.93±17.52 | 0.40 | 7.97% |
|  |  | Comparative cost consequences – other medical costs | 33.87±13.51 | 0.40 | 6.29% |
|  |  | Comparative cost consequences – non-medical costs | 23.20±13.18 | 0.60 | 4.31% |
| Evidence about drugs | 14.63±10.02 | Quality of evidence | 51.87±17.69 | 0.34 | 7.58% |
|  |  | Expert consensus / clinical practice guidelines | 48.17±17.69 | 0.37 | 7.05% |

**Appendix 14. Results of criteria weighted by the public using the two-step percentile distribution method**


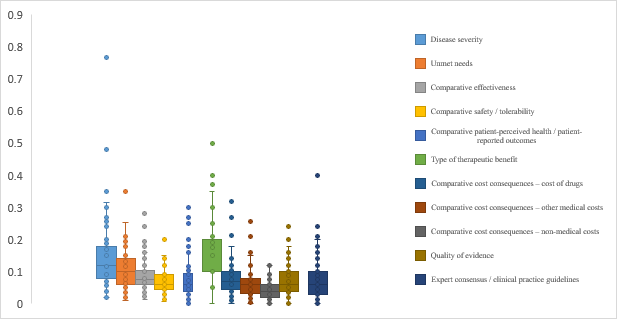

Supplement: Supplementary file 1 — Supplementary Material 1 [file 13023_2024_3393_MOESM1_ESM.docx]
